# Association between vitamin D serum levels and clinical, laboratory, and parasitological parameters in patients with malaria from an endemic area of the Amazon

**DOI:** 10.1590/0037-8682-0077-2021

**Published:** 2022-04-08

**Authors:** Janaina Maria Setto, Rosana Maria Feio Libonati, Ana Maria Revoredo da Silva Ventura, Tânia do Socorro Souza Chaves, Carina Guilhon Sequeira, Arnaldo Jorge Martins, Ricardo Luiz Dantas Machado, Sylvia do Carmo Castro Franceschin, Jéssica Thuanny Teixeira Barreto

**Affiliations:** 1 Marinha do Brasil, Centro de Instrução Almirante Braz de Aguiar, Belém, PA, Brasil.; 2 Universidade Federal do Pará, Núcleo de Medicina Tropical, Programa de Pós-Graduação em Doenças Tropicais, Belém, PA, Brasil.; 3 Instituto Evandro Chagas, Laboratório de Ensaios Clínicos em Malária, Ananindeua, PA, Brasil.; 4 Universidade Estadual do Pará, Centro de Ciências Biológicas e da Saúde, Belém, PA, Brasil.; 5 Universidade Federal do Pará, Centro Universitário do Estado do Pará, Belém, PA, Brasil.; 6 Universidade Estadual do Pará, Centro de Ciências Biológicas e da Saúde, Departamento de Saúde Integrada, Belém, PA, Brasil.; 7 Instituto Evandro Chagas, Seção de Patologia, Ananindeua, PA, Brasil.; 8 Universidade Federal Fluminense, Programa de Pós-Graduação em Microbiologia e Parasitologia, Rio de Janeiro, RJ, Brasil.; 9 Universidade Federal de Viçosa, Programa de Pós-Graduação em Ciência da Nutrição, Viçosa, MG, Brasil.; 10 Escola Superior da Amazônia, Pós-Graduação em Nutrição Esportiva e Estética, Belém, PA, Brasil.

**Keywords:** Malaria, Parasitemia, Vitamin D

## Abstract

**Background:**

Some studies have suggested the importance of vitamin D [25(OH)D] in malaria clinical practice. The prevalence of 25(OH)D deficiency in the Amazon population is not well known, and there are few studies in patients with malaria. This study aimed to evaluate 25(OH)D serum levels in patients with malaria and determine their relationships with epidemiological, clinical, laboratory, and parasitemia data.

**Methods:**

An analytical cross-sectional study of 123 patients with malaria and 122 individuals without malaria was performed in Itaituba, Pará, Brazil, from January 2018 to October 2019, by evaluating sociodemographic, clinical-epidemiological, parasitological, and laboratory data and adopting a 5% significance level. Parametric tests (Student's *t-*test), non-parametric tests (Mann-Whitney *U*), and Spearman’s correlation ([r_s_], for non-parametric variables) were used according to the nature of the distribution of the variables. For the qualitative variables, Pearson's chi-square test, Fisher's exact test, and the G test were used. Spearman's correlation was used to compare the results of the 25(OH)D levels and blood counts performed among patients and the control group.

**Results:**

Malaria patients residing in a mining area had 25(OH)D serum levels that were significantly lower than those in the control group residing in the mining area, though both were within normal levels. Red blood cell counts had an inverse correlation with parasitemia (*Plasmodium falciparum*), and platelet levels had an inverse correlation with parasitemia (*Plasmodium vivax*). 25(OH)D deficiency was evidenced in Itaituba, in the state of Pará, which is an endemic area of malaria in the Amazon region.

## INTRODUCTION

There has been a global reduction in the estimated number of malaria cases: 231 million in 2017 to 218 million in 2018^1^. Brazil has followed this trend, with a 19.1% reduction in malaria cases (194,573 in 2018 to 157,457 in 2019)^2^.

Cusick *et al*.^3^ found an association between vitamin D [25(OH)D] levels and severe malaria in children from Uganda in Africa; they stated that malaria severity would likely be reduced by 9.0% for every 1 ng/mL increase in 25(OH)D plasma levels.

In malaria, hematological exams must be evaluated and interpreted according to patients’ clinical manifestations and by taking into consideration the type of *Plasmodium*, parasitemia, and the degree of immunity^4^ ^-^ ^7^. 

In the Amazonian population, the infectious disease with the greatest impact is malaria^8^. The prevalence of 25(OH)D deficiency and its relationship with malaria is still poorly understood. Therefore, this study aimed to assess the serum levels of this vitamin and its correlation with epidemiological, clinical, laboratory, and parasitemia parameters.

## METHODS

A cross-sectional analytical study of patients with a positive diagnosis for malaria (thick blood smear) was conducted in Itaituba, a municipality located in the southwest region of the state of Pará (PA), in the Amazon region of Brazil^9^.

Patients with suspected malaria who sought help from the Malaria Diagnostic Service located at the Itaituba Municipal Hospital were invited to participate in the study. The study was approved by the Ethics and Research Committee (Comitê de Ética e Pesquisa - CEP) of the Tropical Medicine Nucleus (NMT) of the Federal University of Pará (UFPA) and the Evandro Chagas Institute (IEC). Each patient signed an informed consent form upon acceptance to the study and in accordance with the principles of Declaration of Helsinki, 1964, as revised in 1975, 1983, 1989, 1996, and 2000. The participants were divided into two groups: individuals with a positive thick blood smear test for malaria, and individuals with a negative thick blood smear test for malaria (control). 

All thick blood smears were read twice: once by a microscopist from the Malaria Diagnostic Service of the Hospital Municipal de Itaituba, PA, and then by a microscopist (blinded to the first thick blood smear diagnosis) from the Laboratory of Clinical Trials in Malaria of the IEC in Ananindeua, PA.

The inclusion criteria for the cases were as follows: individuals of both sexes, aged 19 to 59 years, with a positive thick blood smear test for any species of *Plasmodium*, and living in the city of Itaituba, Pará. For the controls, the same criteria were adopted, but only those with a negative diagnosis for malaria (thick blood smear) and who were asymptomatic were included (these people were companions or family members of the malaria patients).

Those who were receiving supplementation of 25(OH)D, calcium, anticonvulsants, corticosteroids, heparin, isoniazid, antihistamine, cholestyramine, or rifampicin were excluded from both groups, as were individuals with alterations in their renal function tests (urea and creatinine), those with chronic renal failure, those with severe malaria (cerebral, severe anemia, or respiratory and renal complications), and pregnant women. 

For the sample calculation, the values for 25(OH)D deficiency and prevalence in individuals with and without malaria (30% and 15%, respectively) were based on the study by Cusick *et al*.^3^ and on the incidence of malaria (2,521 cases) in the municipality of Itaituba, Pará from the year 2015^10^; the two-sample proportions test was used for these calculations. The power of this test was 80% and the alpha level was 0.05, which provided a required “n” of 121 patients in each group (patients and controls). 

In this study, 25(OH)D deficiency was established as a dependent variable, whereas the patients’ sociodemographic data (sex, ethnicity, education, marital status, residence in a mining area, and age) were established as independent variables for the malaria group and the control group. 25(OH)D deficiency was established as an independent variable when related to the malaria variables (*Plasmodium* species, parasitemia, clinical symptoms, history of infection, time for current illness to develop, and time since last malaria episode) as dependent variables.

The identification of *Plasmodium* in the peripheral blood was performed using the gold standard thick-drop technique for the diagnosis and monitoring of malaria treatment^11^.

A 10 mL amount of venous blood was collected from patients with malaria and from the control group. Part of this venous blood (3 mL) was used to acquire the EDTA blood count (analysis via fluorescent flow cytometry with processing using a SYSMEX XS1000i hematology analyzer), at the Itaituba Municipal Hospital in Pará. The remaining 7 mL was centrifuged to obtain serum, followed by preparation in aliquots for storage (-20ºC) and performing the 25(OH)D examinations (Immunoenzymatic/Fluorescence Test, VIDAS® kit, and BIOMÉRIEUX (2015)^12^) and liver function tests, which were performed in the Biochemistry Laboratory at the IEC in Ananindeua, Pará. 

The normal serum levels adopted for the laboratory parameters included:

1. Blood counts (male/female)^13^

- Red blood cells: 3.80-6.50 million/mm³; hemoglobin: 11.5-17.0 g/dL; hematocrit: 37-54%
- Leukocytes: 4,000-10,000/mm³
- Platelets: 150,000-500,000/mm³

1. 25(OH)D level: >20 ng/mL^14^

A clinical score measured the presence and intensity of the signs and symptoms, such as fever, chills, headache, sweating, coughing, dyspnea, abdominal pain, diarrhea, nausea, vomiting, anorexia, oliguria, choluria, arthralgia, myalgia, backache, dizziness, and prostration. Its intensity was graded from 0 to 3 points as follows: 0=absent, 1=mild (somewhat bothersome), 2=moderate (bothersome and partially limits daily activities), and 3=intense (quite bothersome and prevents activities form being performed). 

A Data Collection Protocol was developed to record the participants’ information, which was completed by the members of the study team and later presented (double entry) in a *Microsoft Office Excel* (2007) spreadsheet. Descriptive and analytical statistics were extracted using *Epi Info 3.5.2* (2010), *BioEstat 5.0* (2007), and *SPSS Statistics 17.0* (2010). 

The normality of the quantitative variables (age, parasitemia, time since last infection, time for current illness to develop, 25(OH)D level, and blood count) was evaluated via the D’Agostino-Pearson test (K samples). Parametric tests (Student's *t*-test), non-parametric tests (Mann-Whitney *U* (MW)), and Spearman’s correlation ([r_s_], used for non-parametric variables) were used according to the nature of the distribution of the variables. To interpret the magnitude of the correlation coefficients, the following values were used: r_s_=0.10-0.30 (weak), r_s_=0.40-0.6 (moderate), and r_s_=0.70-1 (strong)^15^. For the qualitative variables, Pearson's chi-square test, Fisher's exact test, and the G test were used.

Spearman's correlation was used to compare the 25(OH)D level and blood count results among patients and the control group. All study participants had access to the results of the laboratory tests. Individuals with altered exams received referrals and guidance to medical care at the Family Health Strategy Center in Itaituba, Pará.

This study was approved by the CEP of the NMT at the UFPA (CEP decision no. 2.420.183 on 06/12/2017); and the CEP of the IEC in Ananindeua, Pará (CEP decision no. 2.974.069 on 22/10/2018). All participants signed the informed consent form (Resolution 466/2012 of the National Health Board of the Brazilian Ministry of Health).

## RESULTS

There were 245 individuals included in this study (123 with malaria and 122 without), which was conducted between January 2018 and October 2019 in the municipality of Itaituba, Pará, Brazil. Table 1 shows the sociodemographic characteristics of the sample. The patients with malaria had a mean age (33.7±9.9 years) that was significantly lower than that of those without malaria (39.9±9.7 years; *p*<0.001, *t*-test). The cases caused by *Plasmodium vivax* (113 out of 123, or 91.9%) predominated, followed by *Plasmodium falciparum* (10 out of 123, or 8.1%). Among malaria patients, 18.7% (23 of 123) were experiencing their first episode of malaria. Most (100 of 123, or 81.3%) reported previous episodes of malaria (one episode: 20 patients; two or more episodes: 80 patients). The median (interquartile range, IQR) time since the first symptom in the current illness was 4±5 days. The median time since the last malarial infection was 3±15.5 months (median±IQR).

**TABLE 1:** Sociodemographic characteristics of the malaria and control cases (Itaituba, Pará, Brazil, 2018-2019).

Variable	Case 123	Control 122	***p*-value**
	n (%)	n (%)	
**Sex**			
Male	86 (69.9)	58 (47.5)	<0.001*
Female	37 (30.1)	64 (52.5)	
**Ethnicity**			
White	18 (14.6)	27 (22.1)	1.00***
Brown	85 (69.1)	82 (67.2)	
Black	20 (16.3)	13 (10.7)	
**Education**			
Higher education	2 (1.6)	26 (21.3)	1.00***
Postgraduate	-	4 (3.3)	
High school	39 (31.7)	46 (37.7)	
Elementary school	82 (66.7)	45 (36.9)	
Illiterate	-	1 (0.8)	
**Marital status**			
Married / Domestic partnership	69 (56.1)	73 (59.8)	1.00***
Separated/divorced/widowed	4 (3.3)	6 (4.9)	
Single	50 (40.7)	43 (35.2)	
**Residing in the mining area**			
Yes	119 (96.7)	31 (25.4)	<0.001**
No	4 (3.3)	91 (74.6)	

*Pearson's chi-square test; **Fisher's exact test; ***G test.

The malaria triad (fever, chills, and headache) occurred in 55.3% (68 out of 123) of the patients. The most frequent and most intense clinical symptoms were headache, back pain, fever, inappetence, chills, arthralgia, myalgia, asthenia, abdominal pain, sweating, and dizziness, which, taken together, culminated in an intensity clinical score between 4 and 44 points (14±10.5 points).

### Parasitemia

There was no difference between the median values of parasitemia for either *P. vivax* (3,500±6,000 parasites/mm³) or *P. falciparum* (1,500±2,850 parasites/mm³; *p=*0.10, MW test). A history of previous malaria infection (none, one, two or more) had no influence on the parasitemia caused by *P. vivax* (*p=*0.91, *t*-test) or *P. falciparum* (*p=*0.51, MW test). The mean value for the *P. vivax* parasitemia (7,789±9,199 parasites/mm³) in the patients who reported the malaria triad was different from those who did not report it (5,028±7,749 parasites/mm³; *p=*0.09, *t*-test). Likewise, the median value of the *P. falciparum* parasitemia (165±3,065 parasites/mm³) in the patients who reported the malaria triad was not significant in relation to those who did not report it (2,000±1,500 parasites/mm³; *p=*0.19, MW test). 

There was no difference in the parasitemia means of the patients with *P. vivax* malaria and low 25(OH)D levels (6,656±9,242 parasites/mm³) compared to patients with *P. vivax* malaria and normal 25(OH)D levels (6,565±8,488 parasites/mm³; *p=*0.96; *t*-test). In the patients with *P. falciparum* malaria and low 25(OH)D levels, the mean value of parasitemia (4,250±2,475 parasites/mm³) was not statistically different compared to patients with *P. falciparum* and normal 25(OH)D levels (10,815±27,974 parasites/mm³; *p=*0.76; *t*-test). There was also no correlation between clinical scores and *P. vivax* or *P. falciparum* parasitemia (r_s=_0.037 and *p=*0.70, and r_s=_-0.030 and *p*=0.93, respectively; Spearman's correlation).

### 25(OH)D

The serum concentrations of 25(OH)D were within normal levels (>20 ng/mL) in the malaria and control groups, with mean values of 32.3±11.9 ng/mL and 34.7±11.5 ng/mL, respectively, and with no significant difference among the groups (*p=*0.11, *t*-test). Low serum 25(OH)D levels were seen in 28.5% (35 out of 123) of the patients with malaria and in 24.6% (30 of 122) of the control group, with no significant difference (*p=*0.58, Pearson's chi-square test). 

The patients with malaria residing in the mining area had a mean 25(OH)D value (32.2 ng/mL) that was significantly lower than the controls residing in the mining area (38.3 ng/mL; *p=*0.01; *t*-test), however, both values were within the reference range. In terms of the plasmodial species involved (*p=*0.42, Fisher's test), there was no statistical difference between the frequency of low serum levels of 25(OH)D in individuals infected with *P. vivax* malaria (29.2% (33 out of 113)) compared to individuals infected with *P. falciparum* malaria (20% (2 out of 10)). 

Table 2 shows the non-significant comparisons among patients with normal and low serum levels of 25(OH)D in terms of the following variables: history of malaria, malaria triad, time for current illness to develop, time since last bout of malaria, and clinical score.

**TABLE 2:** Clinical-epidemiological parameters of the malaria cases (Itaituba, Pará, Brazil, 2018-2019).

	Normal serum levels	Low serum levels of	
Variable	of 25(OH)D	25(OH)D	p-value
	n (%)	n (%)	
History of malaria:			
- First infection	17 (19.3)	6 (17.1)	0.95*
- 1 prior infection	14 (15.9)	6 (17.1)	
- 2 or more prior infections	57 (64.8)	23 (65.8)	
Malaria triad	47 (53.4)	21 (60.0)	0.64**
Time for current illness to develop (days)	4±3	4±2	0.29***
Time since last infection (months)	2±11	10.5±46	0.10***
Clinical score (points)	16±7	14±8	0.31****

*G test, **Pearson's chi-square test, ***Mann-Whitney U test, ****Student's t-test.

### Hematological parameters

Table 3 shows the hematological parameters of the patients with malaria and those of the control group. A prior history of malaria was significantly related to serum levels of leukocytes. Patients infected for the first time had a lower median serum level of leukocytes (4,734 mm^3^) than patients with a prior history of malaria (1 episode: 6,215 mm^3^; 2 or more episodes: 6,237 mm^3^; *p=*0.004, MW test). A history of malaria showed no statistical difference in relation to the serum levels of red blood cells (*p=*0.93, *t*-test).

**TABLE 3:** Biochemical parameters for the malaria and control cases (Itaituba, Pará, Brazil, 2018-2019).

Variable	Case 123	Control 122	p-value
25(OH)D (ng/mL)	32.3±11.9	34.7±11.5	0.11*
Blood count			
HEM (million/mm^3^)	4.9±0.6	4.7±0.6	0.02*
Hb (g/dL)	14.0±1.7	13.8±1.7	0.35*
Ht (%)	41.5±5.9	40.9±5.3	0.42*
LEU (mm^3^)	5.400±3.000	6.150±2.700	0.06**
PLA (mm³)	139.236±82.003	274.073±94.809	<0.001*

**25(OH)D:** vitamin D; **HEM:** red blood cell count; **Hb:** hemoglobin; **Ht:** hematocrit; **LEU:** leukocytes; **PLA:** platelets. *Student's t-test; **Mann-Whitney U test.

The time (days) for the current illness to develop showed an inverse correlation (albeit weak) with the serum levels of platelets (r_s_=-0.258 and *p=*0.004; Spearman's correlation). The time for the current illness to develop (days) was not correlated with the serum levels of red blood cells or hemoglobin (r_s_=-0.063 and *p=*0.5, and r_s_ =-0.128 and *p=*0.16, respectively; Spearman's correlation).

In this series, the mean number of red blood cells was significantly higher in the group with malaria than in the control group. However, when the serum levels of red blood cells were stratified by sex, there was no statistical difference (*p=*0.83; *t*-test) between the cell counts from men with malaria (4.99±0.56 million/mm^3^) and from the men in the control group (5.01±0.62 million/mm^3^). Among the women, the mean number of red blood cells in the group with malaria (4.52±0.54 million/mm^3^) and in the control group (4.36±0.47 million/mm^3^) also showed no statistical difference (*p=*0.12; *t*-test). However, when stratifying by diagnosis, the men in the malaria group had a mean number of red blood cells (4.99±0.56 million/mm^3^) that was significantly higher in than the women (4.52±0.54 million/mm^3^; *p*<0.001; *t*-test). The same occurred in the control group, in which the median value for the number of red blood cells among men (5.01±0.90 million/mm^3^) was significantly higher than of the women (4.30±0.58 million/mm^3^; *p<*0.001; MW test).

Correlations between the study variables in patients with malaria (Table 4)

**TABLE 4:** Predictors of red blood cell and platelet counts in the malaria cases (Itaituba, Pará, Brazil, 2018-2019).

Case 123			
	Variables	r_s_	***p*-value**
Red blood cells	Parasitemia (*P. vivax*)	0.061	0.52
	Parasitemia (*P. falciparum*)	-0.659	**0.04**
Platelets	Parasitemia (*P. vivax*)	-0.331	**<0.001**
	Parasitemia (*P. falciparum*)	-0.365	0.30

**rs:** Spearman's correlation coefficient.

In the patients with *P. falciparum* malaria, there was a moderate inverse correlation between parasitemia and the number of red blood cells: the greater the parasitemia, the lower the serum levels of the red blood cells (*r*_*s*_ =-0.659, *p=*0.04; Spearman's correlation). Among the patients with *P. vivax* malaria, there was an inverse correlation (albeit weak) between parasitemia and platelets: the greater the parasitemia, the lower the platelet serum levels (*r*_*s*_ =-0.331, *p=*0.04; Spearman's correlation). 

## DISCUSSION

This study evaluated the serum levels of 25(OH)D in patients with malaria and its relationship with epidemiological, clinical, laboratory and parasitemia parameters at the Malaria Diagnostic Service, which is located in the Municipal Hospital of Itaituba, Pará, Brazil. Low serum levels of 25(OH)D were identified among individuals with malaria (28.5%) and without malaria (24.6%), but without statistical difference. However, serum platelet levels showed a significant inverse correlation with parasitemia (*P. vivax*) and with the period of time since the onset of the disease. In addition, red blood cells showed a significant inverse correlation with parasitemia (*P. falciparum*). Patients with primary malaria infection had significant leukopenia.

Among the patients with malaria, the male gender predominated (69.9%); this is similar to the results reported (71.9%) by Lopes *et al*. ^16^, who conducted a study in the same location as this present study (the municipality of Itaituba, Pará, Brazil). Economic mining exploration (such as in Itaituba) is mainly performed by men^9^ ^,^ ^17^ ^-^ ^20^. Thus, malaria is strongly associated with professional activities in various parts of Brazil^21^, occurring more frequently among men^22^ ^,^ ^23^. Most of the individuals with malaria (96.7%) stated that they resided in mining areas. Significant alterations in the environment resulting from the exploration of natural resources such as diamonds and/or gold, wood, and grains trigger an ecological imbalance, thereby facilitating the interaction of susceptible people and the vector transmitted by *Plasmodium* ^23^ ^,^ ^24^.

Regarding education, most of the patients with malaria had little education: 66.7% had only elementary education, which is in accordance with other Brazilian studies^16^ ^,^ ^25^. According to Catraio *et al*.^26^, there is a direct proportional relationship between an individual's level of education and the health-disease process. 

In the group with malaria, the mean age was 33.7±9.9 years; this is a range that includes the economically active age group of the population and is a result that is similar to that reported by other authors^16^ ^,^ ^27^.

In Brazil, the main species causing malaria is *P. vivax* ^8^, which predominated in this study (91.3%); this result was similar to that observed by other authors in Brazilian studies^16^ ^,^ ^21^ ^,^ ^25^. 

The prevalence of low serum levels of 25(OH)D were similar in both groups: 28.5% in the patients with malaria and 24.6% in the control group. However, the patients with malaria who resided in the mining area had lower 25(OH)D levels than patients in the control group that also resided in the mining region, although the levels in both groups were within the normal range. The association between low serum levels of 25(OH)D and malarial infection has been verified in the results of authors highlighting the role of 25(OH)D in experimental malaria^28^ ^,^ ^29^ and in children with severe malaria^3^.

In a study of 60 children (aged 18 months to 12 years) in Uganda, Africa, in which 40 had severe malaria (severe anemia or cerebral malaria) and 20 did not have malaria, a high percentage of 25(OH)D insufficiency (<30 ng/mL) was found in both groups (95% and 80%, respectively), with significantly reduced levels of 25(OH)D in those with severe malaria. Although the authors emphasized that their results were preliminary due to the need for a larger sample size of children in order to analyze the relationship between 25(OH)D levels and malaria severity, upon using a logistic regression model, the authors concluded that for each 1 ng/mL increase in 25(OH)D plasma levels, the chance of having severe malaria declined by 9%^3^.

The lack of association between low serum levels of 25(OH)D and malaria determined in this study compared to the results above may be due to the age range of the participants (adults), the predominance of the *Plasmodium* species (91.9% *P. vivax*), and the type of malarial infection (uncomplicated malaria). 

In this series, there was no correlation between parasitemia and a patient’s clinical score, nor between parasitemia and presence of the malaria triad (fever, chills, and headache), which is similar to the results by Silva *et al*.^30^. Therefore, it can be suggested that the patients in this present study have some degree of clinical immunity, given that 81.3% of the patients reported a prior history of malaria; this is in line with a partial status of protection against malaria, which induces oligosymptomatic or even asymptomatic forms of the disease, especially in individuals exposed to repeated *Plasmodium* infections^8^.

Our patients with *P. falciparum* malaria had an inverse correlation between parasitemia and serum levels of red blood cells; this is in contrast to the patients infected with *P. vivax*, who did not demonstrate such a correlation. It is known that both species of *Plasmodium* can cause hemolysis^31^; however, *P. falciparum* is the more virulent species, invading red blood cells of all ages, whereas *P. vivax* preferentially infects the reticulocytes (the young forms of erythrocytes)^32^.

According to the results of other studies, primary malaria infection was associated with a decrease in serum levels of leukocytes^33^ ^,^ ^34^. Leukopenia is observed at the beginning of malaria infection, with transient leukocytosis occurring during the febrile paroxysms, which are related to the parasitic burden^35^ ^,^ ^36^.

In the malaria group, the mean red blood cell count was significantly higher than that in the control group. The time for the current illness to develop, time since last infection, and history of malaria were not associated with serum levels of red blood cells and hemoglobin. These results agree with those by Costa *et al*.^34^, who observed (in the Amazon region) higher levels of red blood cells in patients with non-severe acute *P. vivax* malaria than in controls. However, our study differs from others studies that have observed a decrease in red blood cell, hemoglobin, and hematocrit values in patients with malaria. Additionally, anemia is common in patients with severe *P. vivax* malaria^7^ ^,^ ^37^ ^,^ ^38^. This divergence was because the mean number of red blood cells was significantly higher in men than in women (both patients and controls) and because the majority of participants in the group with malaria were men (69.9%). It is known that red blood cell levels are higher in men than in women, as are hemoglobin and hematocrit levels^39^. This difference may involve the influence of factors such as the androgenic hormone in erythropoiesis and the blood loss that occurs during menstruation in women^40^.

In patients with *P. vivax* malaria, it was observed that the greater the parasitemia and the time for the disease to develop, the lower the serum levels of platelets. Thrombocytopenia is a common pathogenic phenomenon in patients with malaria^34^ ^,^ ^41^, commonly occurring in the pathogenesis of malaria, especially *P. vivax* malaria^7^ ^,^ ^42^. Although the determinant mechanisms of the thrombocytopenia associated with *P. vivax* malaria are not well defined, it is believed that they may be related to coagulation disorders, antibody-mediated platelet destruction, platelet aggregation, oxidative stress, platelet sequestration in the spleen, and alterations in the bone marrow^43^ ^,^ ^44^.

The limitations presented in this study are related to its cross-sectional and analytical experimental design, which could not establish a cause-and-effect relationship between the analyzed factors and low serum levels of 25(OH)D based on nutritional status and nutrient consumption.

It can be concluded that in Itaituba, which is in the state of Pará, which is in the Amazon region, the prevalence of low serum levels of 25(OH)D were similar in the group with malaria (28.5%) and the control group (24.6%). However, the patients with malaria that resided in the mining area had lower 25(OH)D levels than those in the control group that resided in the mining area. The serum levels of platelets had a significant inverse correlation with parasitemia (*P. vivax*) and the time for the disease to develop. The serum levels of red blood cells had a significant inverse correlation with parasitemia (*P. falciparum*). Compared to the patients with a prior history of malaria, the patients with their first infection had significant reductions in their serum levels of leukocytes.
